# Sensors Data Analysis in Supervisory Control and Data Acquisition (SCADA) Systems to Foresee Failures with an Undetermined Origin

**DOI:** 10.3390/s21082762

**Published:** 2021-04-14

**Authors:** F. Javier Maseda, Iker López, Itziar Martija, Patxi Alkorta, Aitor J. Garrido, Izaskun Garrido

**Affiliations:** 1 Automatic Control Group (ACG), Institute of Research and Development of Processes, Faculty of Engineering, University of the Basque Country (UPV/EHU), 48013 Bilbao, Spain; itziar.martija@ehu.eus (I.M.); aitor.garrido@ehu.eus (A.J.G.); izaskun.garrido@ehu.eus (I.G.); 2 Intenance, RDT Company, 48100 Munguia, Spain; iker.lopez@intenance.com; 3 Engineering School of Gipuzkoa, University of the Basque Country (UPV/EHU), 20600 Eibar, Spain; patxi.alkorta@ehu.eus

**Keywords:** industry 4.0, industrial internet of things, supervisory control and data acquisition system, machine learning

## Abstract

This paper presents the design and implementation of a supervisory control and data acquisition (SCADA) system for automatic fault detection. The proposed system offers advantages in three areas: the prognostic capacity for preventive and predictive maintenance, improvement in the quality of the machined product and a reduction in breakdown times. The complementary technologies, the Industrial Internet of Things (IIoT) and various machine learning (ML) techniques, are employed with SCADA systems to obtain the objectives. The analysis of different data sources and the replacement of specific digital sensors with analog sensors improve the prognostic capacity for the detection of faults with an undetermined origin. Also presented is an anomaly detection algorithm to foresee failures and to recognize their occurrence even when they do not register as alarms or events. The improvement in machine availability after the implementation of the novel system guarantees the accomplishment of the proposed objectives.

## 1. Introduction

Industry 4.0 is used as a synonym for cyber-physical production systems (CPPSs) or cyber-physical systems when applied in the domain of the manufacturing industry [1]. Industry 4.0 is based on the complete digitalization of value chains through data processing technologies, intelligent software and a new view of sensor facilities [2]. The goals of Industry 4.0 are to promote decentralization, interoperability, modularity and real-time capability. Industry 4.0 promises increased flexibility in production facilities and, in this way, promotes mass customization, better quality and improved productivity [3]. In summary, Industry 4.0 will become one of the broadest areas of research in the next decade [4,5].

However, it is well-known that industry has traditionally demonstrated inertia toward technological change. This current stage of technological development for the industrial transformation to the smart factory is becoming faster, and it is expected to achieve unprecedented levels in operational efficiencies [6]. One solution for overcoming an outdated industry may be the development and implementation of retrofitting techniques with the addition of new technologies for an efficient re-use of existing equipment [7].

Using the Internet and CPPSs, manufacturing plants can be modernized, transforming them into intelligent factories, characterized by a continuous and instantaneous intercommunication between different workstations that make up the production chains [8]. The Industrial Internet of Things (IIoT) and its related domains—industrial networks, big data and cloud computing—will bring great opportunities for promoting these industrial upgrades [9].

Supervisory control and data acquisition (SCADA) systems are the underlying monitoring and control strategy components of CPPSs [10]. The SCADA system supervises a plant or process through a master terminal unit (MTU) with one or more remote terminal units (RTUs). Through the human–machine interface (HMI), users obtain information in real time that allows them to improve decision-making procedures. The SCADA systems’ increasingly efficient connection with the Internet, as well as with corporate networks, allows for the management of a great amount of data [10].

The collection of data generated by the sensors contributes to the improvement of the control and self-diagnostic capacity in different technological areas [11,12,13]. The virtual replication of a manufacturing chain and the improvement of operative procedures and test simulations are the new possibilities for sensors in Industry 4.0 [12,13,14]. The data ingestion of heterogeneous sensors and devices is a challenge which plays a significant role in SCADA systems. Different solutions have been proposed, from the use of device templates to strategies for data synchronization, data slicing, data splitting and data indexing [15,16]. The combination of data mining and analytics with machine learning (ML) algorithms can be used for information extraction, data pattern recognition and fault prediction [17,18].

The evolution of technology and equipment in modern industry has made maintenance tasks more complex and functional. Two tasks can be highlighted in the new generation of SCADA systems: managing a huge amount of different data and fault diagnosis and prognosis [19]. The manufacturing industry generates more data than any other sector of the economy; however, on many occasions, these data do not result in knowledge [20]. One of the main objectives of maintenance is to integrate in SCADA systems diagnosis modules for detecting abnormal situations from sensor data [21]. The alarms and event logs are the base knowledge for analyzing the root cause of most of the failures, something usually undervalued in industry. The ML applications in maintenance tasks can cover these gaps and take advantage of all these data logs, resulting in smarter and more robust manufacturing [22,23,24].

In this study we analyzed the roles that sensor data analysis and treatment play in a SCADA system for fault diagnosis, specifically for faults that have a difficult prognosis due to root causes of unknown origin. Applications of different maintenance procedures for identifying error patterns and estimating the useful life of the machinery components were developed. The fulfillment of the main objective proposed and its application rely on the improvement of the availability of machine tools in chip removal machining processes and its adaptability to other machine processes.

Chip removal machinery are widely used industrial equipment for which the IIoT and ML techniques can notably help to improve the resolution of different maintenance failures in relation to their undetermined root causes.

The methodological approach is based on the challenge of foreseeing faults with an undetermined origin. The first step is the development of a preventive and predictive maintenance procedure integrated in the SCADA system. This procedure analyzes the sensor and actuator database from three data sources: retrofitting, digital twin and event SCADA screens. The IIoT physical support, which facilitates data collection from different sources within a diverse operational environment, is an Open Platform Communications (OPC) system [25,26]. The developed environment is a response to the majority of failures, as their origin becomes known [27,28]. However, some faults and their root causes remain unknown [29]. The analysis of these abnormal situations leads to the decision to change from digital to analog sensors in specific machine variables. The novel features added to the anomaly detection procedure improve fault prognosis. Finally, for resolving hidden faults, fundamentally those related to the malfunctioning of the machine without generating an alarm or event, an unsupervised anomaly detection algorithm is used.

The remainder of this work is organized as follows. Section 2 surveys the related work. Section 3 details the SCADA system’s main functions for failure detection, and provides a global vision of applicable procedures for their resolution. Section 4 presents the proposed IIoT architecture and the protocols implemented. Section 5 sets out the data sources for fault detection. Section 6 describes the use of analog sensors as a solution for the detection of faults with unknown origins. In Section 7, the data management and machine learning techniques and, in particular, an anomaly detection algorithm, are developed. Finally, Section 8 presents the results and conclusions.

## 2. Related Work

This section describes and contrasts related work from the fields of fault and anomaly detection in Industry 4.0.

The authors of [3] have provided a review of intelligent manufacturing techniques in the context of Industry 4.0. In recent years, the explosive growth of sensor data in SCADA systems through IIoT platforms has resulted in an increased use of data mining and analytics in industrial processes, improving the role of machine learning [10,18]. In turn, the authors of [19] surveyed the different artificial intelligent techniques for the fault diagnosis of rotating machinery. A data-driven approach based on alarms, events and exploratory data analysis was proposed in [20].

In order to illustrate the efficiency and usefulness of machine learning in smart manufacturing, with regard to fault diagnosis, different algorithms have been proposed in [21,22]. In [23], the authors outlined machine learning in the fault diagnosis of air handling units, based on the comparison between the observed behavior and a set of behavioral patterns generated through various fault conditions.

Furthermore, the authors of [24] undertook a survey on decision-making procedures based on system reliability for improving predictive maintenance in Industry 4.0. This study analyzed the impact that local failures can impose on an entire company.

Also, anomaly detection algorithms in different industry fields have been widely used. In [30], the authors describe a new methodology for the detection of anomalies in industrial components based on the creation of behavior patterns using unsupervised machine learning algorithms such as K-means for clustering. An algorithm based on probability density distribution was used to enhance the cluster patterns. Likewise, an interesting application of an anomaly detection algorithm for early stage-bearing fault diagnosis was presented in [31]. Similarly, machine learning methods for anomaly detection, improving maintenance activities and security in industrial processes techniques were developed in [32]. Finally, in [33], the author used as a case study the detection of known and unknown faults in the automotive industry by acquiring data during production and testing of vehicles. In this work, two-class and one-class classifiers were evaluated.

Most of the described works in fault and anomaly detection compare different machine learning algorithms applied to a specific case study so that the proposals have enough machine data to use these supervised and unsupervised machine learning algorithms. In addition, the different classifiers have been successfully used to detect the anomalies. The proposal presented in this paper is quite different since the main idea relies on using the most effective procedure and a feasible anomaly detection algorithm for industrial applications in a diverse range of chip removal machinery. A second difference is related to the unknown causes of the failures that make the selection of features for the algorithms difficult and can determine the changes in the machine structure, as it is a case of replacing digital sensors with analog sensors. Another difference is the absence of clear data patterns to assist with the fault identification tasks, as these faults either do not generate events or are related to wear and fatigue in components. Finally, the results of the progressive implementation and adaptation of the proposed system provides a knowledge base for continuous improvement.

## 3. Failure Detection Background

**Figure 1:**
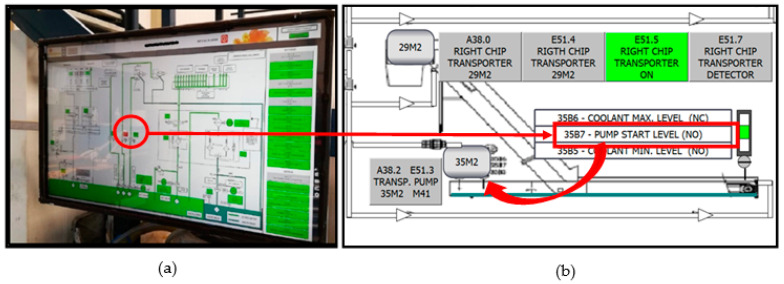
(**a**) Supervisory control and data acquisition (SCADA) system; (**b**) retrofitting scheme as SCADA screen for locating a specific system fault.

Designs and implementations of supervisory control and data acquisition systems for improving the use of industrial machinery have been increasing in recent years, with an evolution towards novel objectives, such as standardization, complex data analysis and robustness against failure [2,21]. Figure 1 shows a SCADA system for controlling and monitoring an industrial system.

Four basic functions performed by SCADA systems for creating autonomous environments with the final objective of the improvement of a fault prognostic for faults with no clear root causes are described below.

Automation: the control and data are linked, in a continuous and reliable way, with the field equipment. Supervision: based on the data movement, the state of actuators and sensors are monitored from an HMI and stored in the Cloud. Alarm management: the events and alarms caused by an abnormal state of the process are gathered. Report generation: the SCADA functions generate a large amount of heterogeneous data, the analysis of which leads to different maintenance procedures, some of them based on artificial intelligent technologies.

Figure 1 shows a typical example of a standard maintenance procedure. In Figure 1a, a fault pops up in a SCADA screen (the red color indicates a fault); in this case, it describes a clogged pump or strainer filter. A sensor detects the fault in a carriage’s hydrostatic lubrication system, a situation difficult to find during a routine inspection. If the user clicks on the SCADA screen fault, the related retrofitting scheme in Figure 1b shows the detection of an anomaly in the starting level of the return pump to the drill tank of a machine’s right chip collector. This level stays at “1”, causing the pump to start and stop constantly, which is not an optimum functioning state for the motor-pump group (35M2).

**Figure 2:**
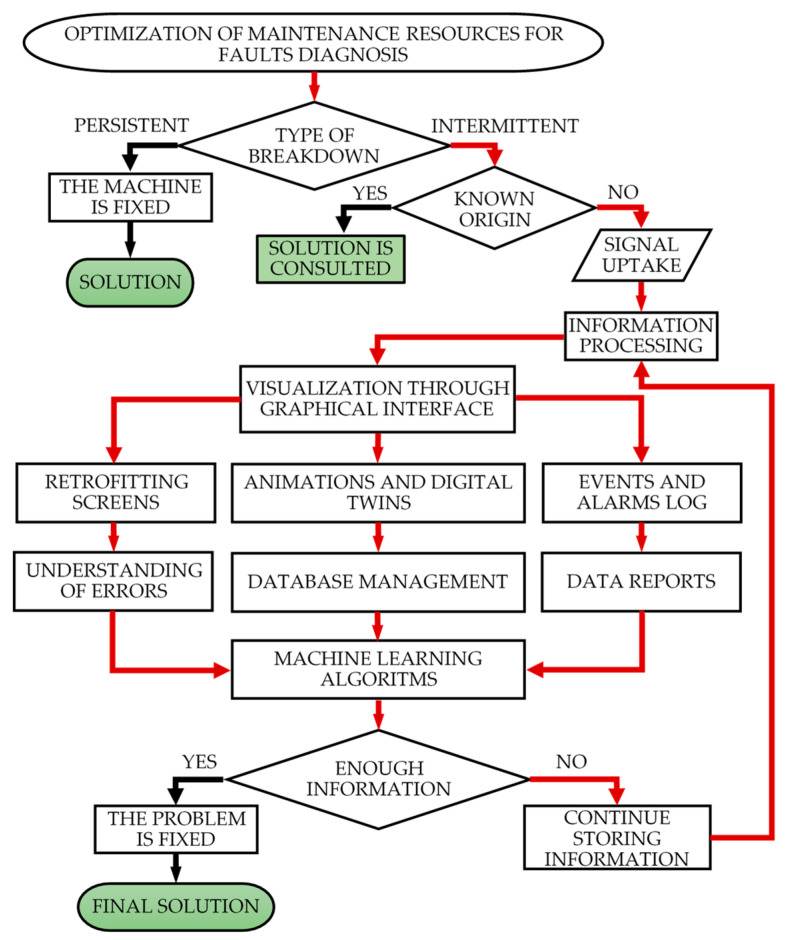
Flowchart for fault diagnosis and resolution.

The proposed SCADA system implements different improvements based on the analysis of data generated in this situation. For example, the number of motor-pump group operations carried out opens new predictive maintenance procedures for monitoring and foreseeing future wear failures in this equipment. However, the question is whether it is feasible to develop a general procedure for the early detection of failures that, on many occasions, may have uncertain origins. Figure 2 shows a flowchart that provides an answer to this question.

The flowchart illustrates the process of analysis of maintenance resource optimization, determining if a breakdown is caused by a persistent or intermittent fault. If the fault is persistent, the machine can be repaired. However, if the fault is intermittent, occurring in erratic sequences with variable durations, it is necessary to define whether the origin of the fault is known or not. When an intermittent problem occurs due to a known fault, the previous procedure is followed: the fault is corrected and the machine re-started, fixing it so it does not occur again.

In cases where the origin of the fault is uncertain, the proposed solution is to be applied. The first thing needed is to know the state of the machine. The next step is to obtain a collection of variables and capture all the signals that intervene in the process for their analysis. Subsequently, all the information collected is processed for visualization in the SCADA graphical interface, created through retrofitting, digital twin and event log screens. The monitoring process does not start when the fault is generated. The application is always in operation in order to collect in real time all the events that occur in the machines.

The continuous processing of these data through different machine learning algorithms can offer sufficient information to solve a failure of which the origin was previously uncertain. The scalability in the use of these algorithms guarantees an optimal management of the fault prognosis [23]. Finally, if the fault diagnosis system still does not have enough information to solve the problem, it will continue collecting data, repeating the process described above.

The last situation relates to the lack of information about the root cause of the problem. These uncertainties are mainly due to the majority of sensors in use being digital. The change from digital to analog sensors in strategic machinery components, which present most of the intermittent failures, can help in identifying the root causes and lead to the development of a solution for them.

## 4. IIoT and SCADA Systems

The Industrial Internet of Things and its related domains, big data and cloud computing, bring great opportunities for promoting industrial advancements in Industry 4.0. The massive data-exchange boosted by SCADA systems and IIoT demands more incisive approaches to the analysis of industrial data. Data mining, analytics support and decision-making play an important new role in the process industry [24]. Analysis of sensor data to predict intermittent failures due to an undetermined root cause belongs within this framework.

**Figure 3:**
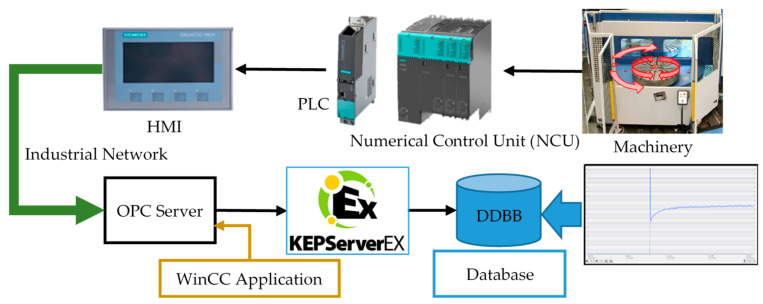
Implemented Open Platform Communications (OPC) scheme and database.

Figure 3 shows the communication platform proposed for linking the SCADA system and the database in the Cloud. However, there are two concepts that should be analyzed: the real-time and the open communication structures. They allow precision and free design for adaptation to different industrial applications [9,10]. The schematic shown in Figure 3 represents a general operation environment, where the procedure for fault diagnosis is integrated and the ML algorithms use the database for prognosis tasks. These IIoT and SCADA systems make data management and the anomaly detection algorithms portable systems with a high degree of independency from the operating environment used for its implementation.

A measuring device that works in real time is able to show the value of a variable at the precise instant when this variable changes its state. When computers, controllers or any device that works based on a computer program to process field information are used, a time lag appears, a delay, which can affect the instantaneous accuracy of the displayed value. This lack of accuracy can go unnoticed, particularly in the measurement of “slow” variables, or it can be considerable in the case of “fast” variables. Figure 3 shows an instantaneous peak of an analog variable that must be accurately recorded and collected in the database.

This delay or time lag can also appear in the devices that make up a telecommunication network, such as switches and routers. Consequently, the term “real time” should also take into consideration these delays in transmission, which could be understood as errors or noise [21].

On the other hand, “real time” is a term that should be properly valued in an industrial environment. It is possible to define the delay that can be tolerated by the process and in this context “strictly in real time” means that a system reacts to external events within that specified time in 100% of cases. If the specific reaction times are overcome without causing relevant problems, as in non-critical systems, it is called “soft real time”.

The main advantage of a PC-based system, its open structure, can become a relevant drawback. The open structure allows the company and developer more freedom in choosing the right tool for the design, programming and implementation of the SCADA system. However, having to centralize the data sent by communication protocols from different manufacturers can generate errors when sending the data and poor analysis from the predictive algorithms. The treatment of corrupted data can be a major inconvenience in obtaining favorable results. The use of Open Platform Communications (OPC) is a solution for this issue.

### 4.1. Open Platform Communications

Open Platform Communications is a communication standard for the secure and reliable exchange of data in the industrial automation environment. It allows the flow of information among devices from different manufacturers. The OPC standard defines the interface between clients and servers, including real-time data, monitoring alarms and events and accessing historical data.

The purpose of this standard is to abstract PLC-specific protocols (such as Profibus, Modbus, Profinet, etc.) into a standardized interface allowing HMI/SCADA systems to convert generic OPC read/write requests into device-specific requests and vice-versa.

With the introduction of service-oriented architectures in manufacturing systems, new challenges in security and data modeling arise. The OPC Foundation developed the OPC UA specifications to address these needs and at the same time provided a feature-rich, open-platform architecture technology that is future-proof, scalable and extensible. This multi-layered approach accomplishes the original design specification goals of functional equivalence, platform independence and security [25].

### 4.2. Software for Interactive Work

Cyber-physical production systems need to include different technologies and have to be able to fulfill the requirements imposed, such as optimization of the manufacturing process, data integration, secure communication and flexible adaptation to changes, among others [1].

SIMATIC WinCC was the software tool used in this project. It is a well-adapted environment for working with SCADA applications. It is a scalable visualization environment endowed with powerful functions for the supervision of automated processes. WinCC provides full SCADA functionality in Windows, from single-user systems to distributed multi-user systems with redundant servers [26].

**Figure 4:**
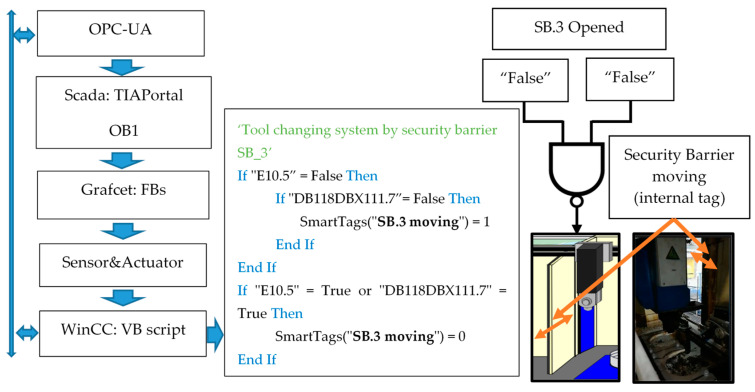
Extract of the VBScript encoded to control an internal security barrier of a machine.

WinCC integrates Visual Basic for Applications (VBA) in WinCC Graphics Designer, a standard environment for application-specific extensions. VBA provides access to all configuration data (variables, warnings, images and graphic objects, with animation included). Figure 4 shows an implementation example.

The result of this software combination, in the HMIs, allows managing users and passwords, comparing different variables, programming several conditions at the same time, sending reports of errors and storage in databases, effectively extending the failure prognosis for most of situations. In the following section, three examples are developed for a better comprehension of the proposal.

## 5. Data Sources for Fault Detection

As previously mentioned, one of the biggest problems faced by developers in the area of industrial maintenance is the lack of knowledge about the exact origin of the failure that causes a breakdown. This results in an indeterminable waste of time—unfortunately more often spent on the search for the failure than its resolution—and therefore in increased production over-costs.

**Figure 5:**
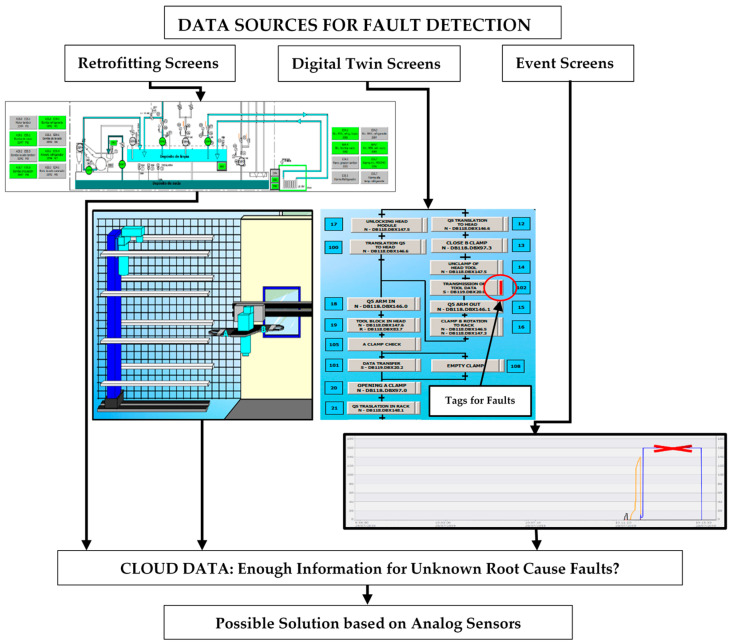
Standard data sources for fault detection.

Information is the solution for fixing the faults and this information is located in the SCADA data. Figure 5 shows three dataset sources for fault detection.

The first source is the data obtained by retrofitting techniques. Figure 5 shows a hydraulics and lubrication screen that has been created and animated from the original static drawing. The retrofitting techniques transform the old documentation into new SCADA screens, which is thus a low-cost solution with optimum results [4]. Drawings of pipes, motors, solenoid valves and so on that change color at the same time that the variables change state immediately indicate if a hose has pressure, if a pump is running or if the machine is projecting coolant while machining. Changes of states in cylinders and electro-valves are also visualized, generating a clearer understanding of the operation of the circuits and helping to instantly identify the causes of a possible fault. In addition, these component signals supply the database for actuators and sensors. Their analysis allows the overcoming of potential failures in components, from intermittent faults to predictions on wear fatigue.

The second data source is obtained from digital twin production systems. Figure 5 shows a virtual twin of a set of automatic tool loaders and its GRAFCET (*Graphe Fonctionnel de Commande Etape Transition).* The digital environment reproduces a real system, its control variables and the functioning conditions: a robot that loads tools from the warehouse, a changing arm that exchanges tools between the machining head and the warehouse robot, the laser detectors for checking and measuring the tools and the different doors that open or close depending on the needs of the machine.

In cases of a failure in a change sequence, the stage tag that has not been completed remains red, alerting the operator where the problem has occurred. The fault is recorded in the database in order to be analyzed, including information on whether the fault is persistent or intermittent and whether its root cause is known.

The third source is the event log. Figure 5 shows a type of screen, perhaps somewhat unusual, for fault detection. The figure shows one specific signal from the total set of events and alarms that appear during the production time. When an anomaly during the machining process happens, the machine activates a series of variables stored in a folder exclusively for events and alarms. By detecting that change of state in these variables, it is possible to visualize what situation is occurring and what part of the system it comes from. Figure 5 also shows an abnormally delayed operation time in a hydraulic cylinder, which could mean a reduction of pressure for its operation, possibly caused by excessive friction or a synchronization fault. The analysis should lead to the root cause prognosis and avoid the foreseeable blockage.

## 6. Analog Sensors as a Solution for Faults with Unknown Origin

Analog sensors provide an opportunity to improve the SCADA system characteristics by increasing their efficiency in predicting failures—in particular, failures that are more difficult to resolve because their origin remains unknown from among many possible root causes. The replacement of a digital sensor with an analog sensor in an industrial context is not an easy decision. Doing so means a modification of the machine structure. In addition, this decision implies that the new feature needs to be included in the fault detection algorithms.

**Figure 6:**
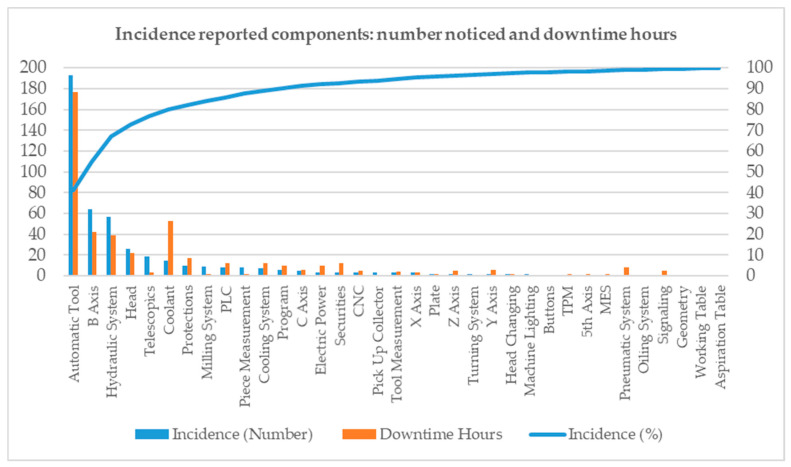
Accumulated number of notices and hours of machinery stoppages.

Figure 6 shows a yearly report of the fault incidence for a specific machine based on the number of notices and downtime hours. The left y-axis shows the number of maintenance notices and downtime hours, in blue and brown bars respectively, for each part of the machine. The Automatic Tool Changer alone accounted for 40% of the machine notices on the right y-axis. Figure 6 illustrates the most critical components in the machine and answers the question as to where to implement the change.

In the case of this particular yearly report, this information led to the decision to change specific digital sensors for analog sensors in these critical components of the machine. This option should be carefully analyzed because it involves physical changes and hardware and software development.

**Figure 7:**
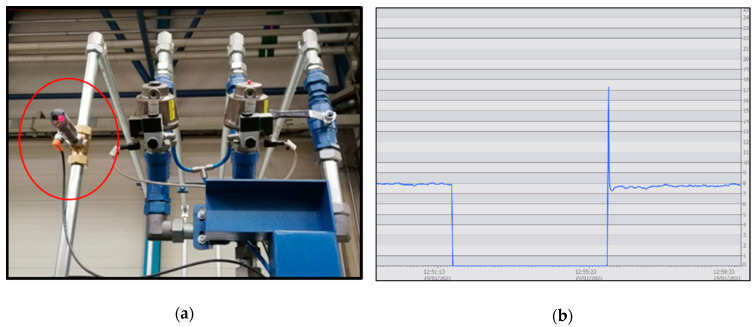
(**a**) Analog flowmeter installed in the machine. (**b**) Coolant flow monitoring.

The real case described in this paper allowed for the analysis of the strategic role of an analog sensor (SA5000 from IFM) installed in the cooling system of a milling machine (Figure 7). The cooling system was the second most common cause of downtime in the machine. The digital flowmeter switch that was previously installed could only indicate if the flow rate of the coolant pump was correct based on a set point programmed in the controller.

After installing the analog flowmeter, the monitoring interface was able to control in real-time the real flow through the coolant pipe. Figure 7a shows the sensor installed in the pipeline and Figure 7b shows a graphic with the pressure flow measurement.

The main goal was to link the information of the cooling system flowmeter with the type of machining operation and tool type used at all times. Then, using all the data extracted from the machine, wear failure of the coolant pumps could be avoided, preventing the machine from being damaged and ensuring the optimal functioning of the cooling systems and the highest quality of the produced pieces.

## 7. Data Management for Improving Prognosis Capabilities and Machine Learning

The Automatic Tool Changer in chip removal machining processes is usually engaged in exchanging milling and drilling tools. It is very important to maintain a stable hydraulic service pressure in this component; a reduction in pressure, for example, causes the tool to vibrate when working and results in roughly machined pieces. This is the problem examined in this section, as it was the most frequent source of notices and downtime hours for the machine described in Figure 6, nearly 40%. For some unknown reason, a drop in pressure can be noticed after certain tool changes. This should not happen due to the existing nitrogen hydraulic accumulator in the pressure circuit. The system must ensure the rotary shaft remains free of blockages in each of the special heads.

**Figure 8:**
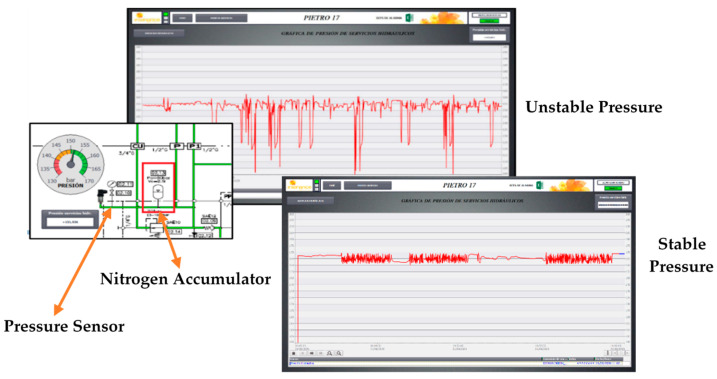
Hydraulic circuit and unstable and stable recorded pressure data.

Figure 8 shows the hydraulic circuit and measured data in the pressure sensor of the analyzed system. The sensor used was the same analog sensor as previously (SA5000 from IFM), which can be programed as an analog flowmeter or an analog pressure meter.

In this kind of maintenance problem, the difficulty of determining the useful life of each element of the hydraulic circuit and the discharge of the nitrogen accumulator is assumed. In addition, the problem involves not only an unexpected machine stop, but also the quality of the machining. This last problem was detected with a product quality inspection but the machine did not give any warning of the event. What is proposed is the application of different techniques to determine whether intervention in the equipment is necessary based on the value of certain indicators. The techniques that can be employed to acquire or determine these indicators can be non-invasive measurements, performance data, scheduled tests and behaviors detected from the monitoring system.

### 7.1. Condition-Based Maintenance

Condition-based maintenance (CBM) combines three elements: data acquisition, data processing and maintenance decision-making [27,28]. A simple procedure for maintenance decision-making is to perform the statistics calculation of SCADA data in intervals in different time horizons and, in this way, check whether the possible anomalous trends detected are the result of some specific requirement or are maintained over time. The statistics calculations chosen for this purpose were the arithmetic mean and the standard deviation.

The arithmetic mean of a finite set of values aims to offer the expected value or the mathematical expectation of the sample used. To do this, all the sample values are added (x^(i)^) and divided by the number of addends (m).

(1) $$\mu =\frac{1}{m}\;\cdot \;\overset{n}{\underset{i=1}{\sum }}x^{\left(i\right)}$$

The standard deviation is a statistic used to quantify the variation or dispersion of a set of data, indicating its tendency to be grouped close to its mean value.

(2) $$\sigma =\sqrt{\frac{1}{m}\;\cdot \;\overset{m}{\underset{i=1}{\sum }}{{(x}^{\left(i\right)}-\mu )}^{2}}$$

**Figure 9:**
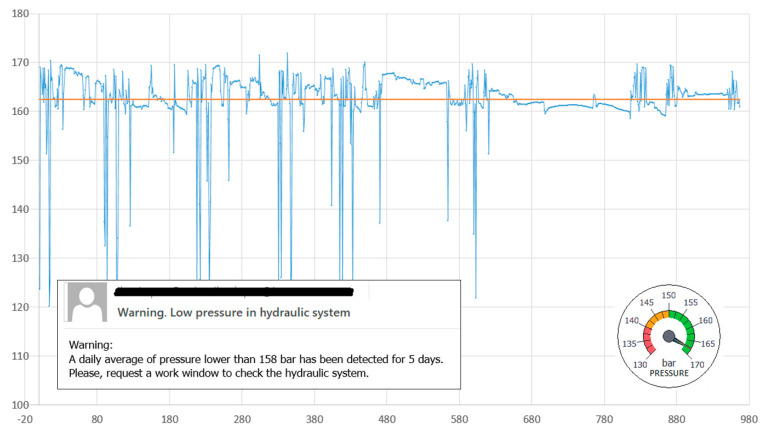
Captured data and the arithmetic mean value under standard conditions.

In Figure 9, pressure data captured over a certain period of time, the calculated arithmetic mean value (165.30 bar) and the standard deviation (2.2821) are shown. These data are considered within the standard work conditions window. If the pressure evolution generates a notice or error in the CDM system, a warning is sent to the maintenance staff.

Under standard conditions, the working pressure of the system is between 150 and 170 bar. The fluctuations are due to the demand pumps and pressure accumulator. The idea is to record the pressure values every 500 ms, whenever the machine is in operation, and to include these data in the statistics method, which would establish the conditions for preventive and predictive maintenance actions.

The value of the arithmetic mean and its evolution can be used as an indicator of the wear of the pump and the accumulator. In our case study, for the detection of a continued decrease over time, a daily average lower than 162 bar was set as a standard, and if maintained over five days it would lead to a request for a time window to intervene in the machine and check the status of the hydraulic system. On the other hand, a standard deviation greater than 3.25 was set to trigger a review notice and activate preventive maintenance; a deviation greater than 3.75 bar was set to trigger a notice for high priority maintenance action.

### 7.2. Machine Learning

Machine learning is a subset of artificial intelligence methods actively used in industrial settings [22]. The applications involve supervised and unsupervised algorithms for supporting the optimization of maintenance decisions about locating faults and machine malfunctioning with an unclear origin [23,24].

A significant issue in the case study under examination is that faults very rarely occur and when they do occur, they depend on multiple factors. The creation of different behavior patterns based on the progressive degradation of industrial components can provide advance failure warning and precise fault detection [29,30,31]. Most of pertinent studies in the literature compare different classifiers when dealing with a specific problem in a machine component, for which the probability of failure is studied or predicted. On many occasions, there are data available that allow the identification of a behavior pattern model that can be considered “normal” or there are anomaly data for training and cross-reference dataset creation [30,31,32,33]. In our case study, the failure or malfunction is detected, but its origin is undetermined and so is the component, or components, of the machine that cause it. In addition, there are no initial data for the anomaly to adjust the metric for predicting the fault. These two facts make it difficult to predict and search for the origin of the failure. In this case study, unsupervised machine learning algorithms for anomaly detection can be employed to investigate possible solutions for these situations.

The data features considered are the pressure (x_1_) and the tool’s three axis positions (x_2_, x_3_, x_4_) for robotic arm movement. Table 1 shows a set of real data for analysis.

The number of the sample *m* for the anomaly detection algorithm can be defined in a flexible mode. As previously mentioned, the sample time for security in the OPC UA is 500 ms; all the features are captured at the same time and, as a result, in sixty seconds the system can have 120 samples for each feature. In the following analysis, m *=* 468.

(3) $$\mathrm{Data}\;\mathrm{set}=\left\{\begin{array}{ccc} x_{1}^{1} & x_{2}^{1}{\;x}_{3}^{1} & x_{4}^{1}\\ \vdots & \vdots \vdots & \vdots \\ x_{1}^{m} & {\;x}_{2}^{m}{\;x}_{2}^{m} & x_{4}^{m} \end{array}\right\}$$

The anomaly detection is based on a Gaussian distribution:

(4) $$p\left({x;\mu ,\sigma }^{2}\right)=\frac{1}{\sqrt{2\pi }\sigma }\exp^{\left(-\frac{{\left(x-\mu \right)}^{2}}{{2\sigma }^{2}}\right)}$$

where µ is the arithmetic mean and σ^2^ is the variance.

The Gaussian density for all features can be determined as follows:

(5) $$p\left(x\right)=p\left(x_{1}{;\mu }_{1}{,\sigma }_{1}^{2}\right)\;p\left(x_{2}{;\mu }_{2}{,\sigma }_{2}^{2}\right)p\left(x_{3}{;\mu }_{3}{,\sigma }_{3}^{2}\right)\ldots .p\left(x_{n}{;\mu }_{n}{,\sigma }_{n}^{2}\right)=\overset{n}{\underset{j=1}{\prod }}p\left(x_{j}{;\mu }_{j}{,\sigma }_{j}^{2}\right)$$

(6) $$p\left(x\right)=\overset{n}{\underset{j=1}{\prod }}p\left(x_{j}{;\mu }_{j}{,\sigma }_{j}^{2}\right)=\overset{n}{\underset{j=1}{\prod }}\frac{1}{\sqrt{2\pi }\sigma_{j}}\exp^{\left(-\frac{{\left(x_{j}{-\mu }_{j}\right)}^{2}}{{2\sigma }_{j}^{2}}\right)}$$

The anomaly detection is activated when the Gaussian density has a lower probability than a constant value “epsilon”:

(7) p(x)<ε  

This value ε is selected based on the F_1_ score metric.

After observing the distribution data, we implemented a multivariate Gaussian distribution:

(8) p(x;μ,Σ)=1(2π)n2|Σ|12exp(−12 (x−μ)TΣ−1(x−μ))

where $\mu \in R^{n}$ is the arithmetic mean vector and $\Sigma \in R^{\mathrm{nxn}}$ is the covariance matrix.

**Figure 10:**
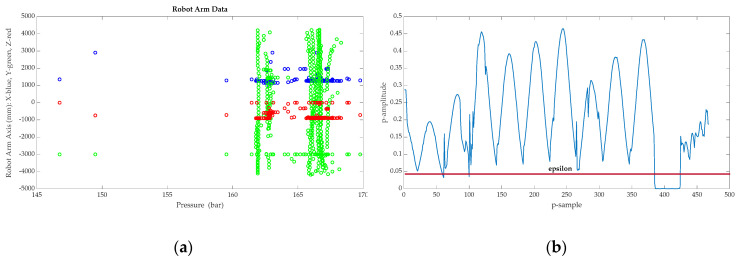
(**a**) The position arm axis related to hydraulic circuit pressure with about m *=* 468 samples for each feature. (**b**) Multivariate Gaussian distribution p(x;μ,Σ).

The results can be observed in Figure 10a,b. Figure 10a shows the data distribution, with the pressure in bars on the x-axis and the three-dimensional robot arm position (XYZ) on the y-axis. Figure 10b shows probability results, analyzed as a multivariate Gaussian distribution.

As previously mentioned, the constant ε for accomplishing p(x)<ε is adjusted through the F_1_ score metric. A point of difference in this case is that, compared with other applications of anomaly detection algorithms, there is no pattern for the recognition of positive faults. This fact makes it difficult to use a cross-reference dataset to adjust the metric. Once the epsilon value has been selected, the applied solution consists in analyzing the possible outliers, which in this case results in 17 situations of possible anomalies that could result in a malfunction in the machining process and consequent quality faults in the machined pieces. These outliers result in *t_p_* (true positives situations), *f_p_* (false positive situations), *f_n_* (false negative situations) and *t_n_* (true negative situations) and this information allows for the improvement of the precision of the F_1_ score metric for future prognosis. The precision (P) and recall (R) parameters are applied for computing the metric:

(9) $$F_{1}-\mathrm{score}=2\frac{\mathrm{PR}}{P+R}$$

P and R can be obtained as follows:

(10) $$P=\frac{t_{p}}{t_{p}+f_{p}}$$

(11) $$R=\frac{t_{p}}{t_{p}+f_{n}}$$

where *t_p_* represents the true positive situations where the prediction and the actual situation are coincident and the fault exists; *f_p_* represents the false positive situations where a fault is predicted but there is no fault in the machine; *f_n_* represents the false negative situations where the prediction is that there is no fault, but the fault exists in the machine; and, finally, *t_n_* represents the true negative situations where the prediction and the actual situation are coincident and the fault does not exist.

## 8. Results and Conclusions

The novel supervisory control and data acquisition (SCADA) system proposed can contribute to advances relevant to the analysis of faults with undetermined origins. This knowledge helps in foreseeing these faults and improving the functionality of machinery.

The main contributions of this work can be summarized in the following six points: firstly, the implementation of a maintenance procedure for fault prognosis integrated in a SCADA system; secondly, the design of a communication platform that linked the SCADA system and a Cloud server for data concentration in a secure and normalized database; thirdly, the development of different data sources for fault detection that were connected to the database; fourthly, fault prognosis was improved when changing from digital to analog sensors; fifthly, the foreseeing of failures with an undetermined origin; finally, the development of an unsupervised anomaly detection algorithm for the detection of malfunctioning machinery.

The results offer advantages in three areas: an increase in prognosis capacity for preventive and predictive maintenance, an improvement in machined product quality and considerable reductions in breakdown times.

**Figure 11:**
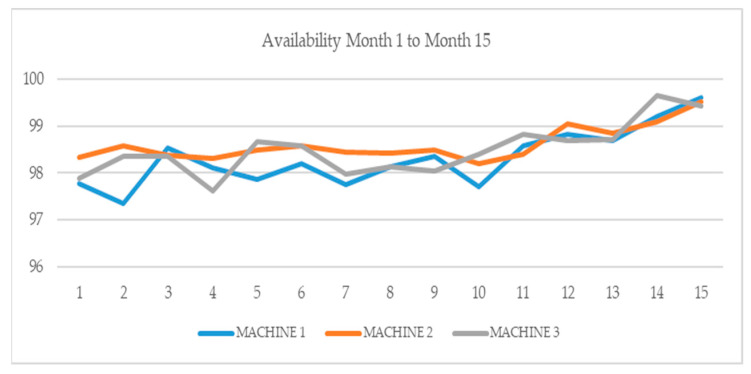
Machine availability evolution.

Table 2 and Figure 11 show the availability improvement of three different machine tools in machining processes of chip removal over fifteen months in 2019 and 2020, after the implementation the proposed system.

The three machines analyzed were in the same production line and each of them had different architectures and tasks. Table 1 represents the data used for training the anomaly detection algorithm for machine 1. Machines 2 and 3 (as seen in Table 2 and Figure 11 together with machine 1) were twin machines that did not have a y-axis. The implementation of the proposed technical solution in these different machines shows a continuing improvement in the availability of the three machines. This demonstrates that this algorithm has potential for widespread implementation in different types of machines.

The progressive improvements in the fifteen-month period can be summarized as follows: in the first five months, retrofitting, digital twin and event screens were created and tested; in the following three months, the OPC UA server and client communication was developed; and from the eighth month onward, machine learning algorithms and data management became operative. It is possible to observe how the improvement was progressive and increased notably over the last five months. The SCADA systems and the machine learning algorithms integrated in them improved their accuracy and efficiency for early fault detection due to the amount of data that they acquired.

One of the most noteworthy points of the proposal presented in this paper was that, for many of the solved problems, their causes could not be analyzed before applying the proposed methodology. The failures occurred but their root causes were not known. As a result of the real examples described throughout the paper, it was possible to show that not only can the origins of problems that cause machine downtime be analyzed, but that the root causes of problems that result in the production of low-quality machined parts can be analyzed as well.

In summary, this study described a SCADA system that combines complexity and agility in the relationship between operator and machine to improve fault prognosis for undetermined root causes. It is recommended that industries continue to research and innovate to obtain the best possible performance outcomes from the potential of the smart factory. The development of more sophisticated SCADA systems to optimize sensor data management and the improvement of industrial predictive maintenance with the implementation of robust diagnosis tools are means to meet this challenge.

The results obtained in this industrial research project, through the investigation of a multi-featured production process affected by intermittent faults and malfunctions of undetermined origins, were applied within the context of Industry 4.0 and the Industrial Internet of Things. The progressive evolution of this work will be centered on the latest research developments concerning the recognition and prognosis of failures that do not generate events when they are occurring. Systematic data analysis of different machines with the same problem and the application of machine learning techniques will result in the development of common procedures for the prediction of these failures that are difficult to detect with standard maintenance operations.

## Figures and Tables

**Table 1:** Input data: sensor pressure (x_1_) and the tool’s three axis positions (x_2_, x_3_ and x_4_).

Pressure (bar)	X-axis (mm)	Y-axis (mm)	Z-axis (mm)
166.7101	1450.00	−3000.8	0
166.577	1450.00	−3000.8	0
166.6435	1612.44	−3000.8	−82.5457
149.4734	2901.50	−3000.8	−737.6
163.0498	2901.50	−3000.8	−737.6
166.4439	2901.50	−3000.8	−737.6
162.9167	2363.06	−3000.8	−463.9846
165.8449	1450.00	−3000.8	0

**Table 2:** Scheduled hours and loss of availability in three machines over fifteen months.

		Mth1	Mth2	Mth3	Mth4	Mth5	Mth6	Mth7	Mth8	Mth9	Mth10	Mth11	Mth12	Mth13	Mth14	Mth15
MACHINE 1	Scheduled Hours	545.99	571.78	550.83	614.76	612.86	547.47	583.15	536.26	586.94	569.5	546.38	528.15	531.06	515.17	264.29
	Loss of Availability (Hours)	12.2	15.17	8.08	11.62	13.09	9.8	13.04	10.05	9.66	13.03	7.79	6.19	6.88	4.12	1.03
	Availability (%)	97.77	97.35	98.53	98.11	97.86	98.21	97.76	98.13	98.35	97.71	98.57	98.83	98.7	99.2	99.61
MACHINE 2	Scheduled (Hours)	552.77	548.7	586.22	649.74	656.2	579.59	602.76	552.53	596.28	587.88	593.31	546.66	561.34	568.02	279.87
	Loss of Availability (Hours)	9.16	8.26	9.54	11.01	9.95	8.18	9.34	8.75	9.07	10.52	9.46	5.27	6.51	5.16	1.35
	Availability (%)	98.34	98.59	98.37	98.31	98.48	98.59	98.45	98.42	98.48	98.21	98.41	99.04	98.84	99.09	99.52
MACHINE 3	Scheduled (Hours)	548.73	549.43	550.97	618.85	580.66	524.3	592.2	548.46	601.08	559.86	558.7	487.97	536.94	560.01	262.54
	Loss of Availability (Hours)	11.64	9.02	9.08	14.75	7.79	7.38	11.97	10.27	11.74	8.97	6.57	6.39	6.89	1.88	1.51
	Availability (%)	97.88	98.36	98.35	97.62	98.66	98.59	97.98	98.13	98.05	98.4	98.82	98.69	98.72	99.66	99.44
		33	32.45	26.7	37.38	30.83	25.36	34.35	29.07	30.47	32.52	23.82	17.85	20.28	11.16	3.89

## Data Availability

The data presented in this study are available on request from the corresponding author. The data are not publicly available due to confidentiality reasons.

## Abbreviations

The following abbreviations are used in this manuscript:

SCADA	Supervisory control and data acquisition
IIoT	Industrial Internet of Things
CPPS	Cyber-physical production systems
HMI	Human–machine interface
OPC	Open Platform Communications
CBL	Condition-based maintenance
ML	Machine learning
